# Clustering and climate associations of Kawasaki Disease in San Diego County suggest environmental triggers

**DOI:** 10.1038/s41598-018-33124-4

**Published:** 2018-11-12

**Authors:** Martin Rypdal, Veronika Rypdal, Jennifer A. Burney, Daniel Cayan, Emelia Bainto, Shannon Skochko, Adriana H. Tremoulet, Jessie Creamean, Chisato Shimizu, Jihoon Kim, Jane C. Burns

**Affiliations:** 1 Department of Mathematics and Statistics, UiT the Arctic University of Norway, Tromsø, 9037 Norway; 2 Department of Pediatrics, University Hospital of North Norway, and Department of Clinical Medicine, UiT the Arctic, University of Norway, Tromsø, 9037 Norway; 3 School of Global Policy and Strategy, University of California San Diego, La Jolla, CA 92093 USA; 4 Scripps Institution of Oceanography, University of California San Diego, La Jolla, CA 92093 USA; 5 Department of Pediatrics, University of California San Diego, La Jolla, CA 92093 USA; 6 Cooperative Institute for Research in Environmental Sciences, University of Colorado, Boulder, CO 80309 USA; 7 Physical Sciences Division, National Oceanic and Atmospheric Administration, Boulder, CO 80305 USA; 8 Department of Biomedical Informatics, University of California San Diego, La Jolla, CA 92093 USA

**Keywords:** Immunology, Atmospheric dynamics, Environmental impact, Paediatric research

## Abstract

Kawasaki Disease (KD) is the most common cause of pediatric acquired heart disease, but its etiology remains unknown. We examined 1164 cases of KD treated at a regional children’s hospital in San Diego over a period of 15 years and uncovered novel structure to disease incidence. KD cases showed a well-defined seasonal variability, but also clustered temporally at much shorter time scales (days to weeks), and spatiotemporally on time scales of up to 10 days and spatial scales of 10–100 km. Temporal clusters of KD cases were associated with strongly significant regional-scale air temperature anomalies and consistent larger-scale atmospheric circulation patterns. Gene expression analysis further revealed a natural partitioning of KD patients into distinct groups based on their gene expression pattern, and that the different groups were associated with certain clinical characteristics that also exhibit temporal autocorrelation. Our data suggest that one or more environmental triggers exist, and that episodic exposures are modulated at least in part by regional weather conditions. We propose that characterization of the environmental factors that trigger KD in genetically susceptible children should focus on aerosols inhaled by patients who share common disease characteristics.

## Introduction

Kawasaki disease (KD) is an acute, self-limited pediatric vasculitis that targets the coronary arteries. If untreated, 25% of children with KD will develop aneurysms of the coronary arteries that may thrombose leading to myocardial ischemia or infarction. Although KD is estimated to affect fewer than 6,000 children in the U.S. each year, the incidence is rising in San Diego County where prospective epidemiologic surveillance has been in place for over two decades. While the average incidence per 100,000 children less than 5 years of age residing in San Diego County was approximately 10 for the decade of the 1990s, the estimate from 2006–2015 was 25.5^1,2^. In contrast in Japan, the country of highest incidence, the attack rate in 2014 was 306/100,000 children less than five years of age^3^. A clear seasonal structure has been defined for Japan and other countries in the northern hemisphere with increased numbers of cases in the winter-spring months, a second smaller peak in the mid-summer, and a nadir of cases in the early fall^4,5^.

Although studies have established that host genetics influence susceptibility and disease outcome, the etiologic agent that triggers KD remains elusive, and extensive searches for an infectious agent have not yielded a consistent pathogen. Several lines of evidence suggest an upper respiratory tract portal of entry for the environmental trigger of KD including inflammation of the tissues of the oropharynx and upper airway and infiltration of the bronchi by IgA-secreting plasma cells^6–8^. An antecedent minor infection associated with immune activation followed by an environmental exposure to a specific trigger have been postulated as a sequence that can induce the vasculitis in genetically susceptible children^9,10^. Modulation of the immune system by administration of high-dose intravenous immunoglobulin aborts the inflammatory response in the majority of patients^11^.

Here we analyze the spatiotemporal structure of KD epidemiology in San Diego County. We link the temporal clustering of KD to local- and regional-scale atmospheric conditions and analyze spatiotemporal clustering of clinical phenotypes and gene expression patterns. The results are consistent with an environmental exposure model that suggests one or more airborne environmental triggers.

## Results

### Seasonality and temporal clustering

**Figure 1:**
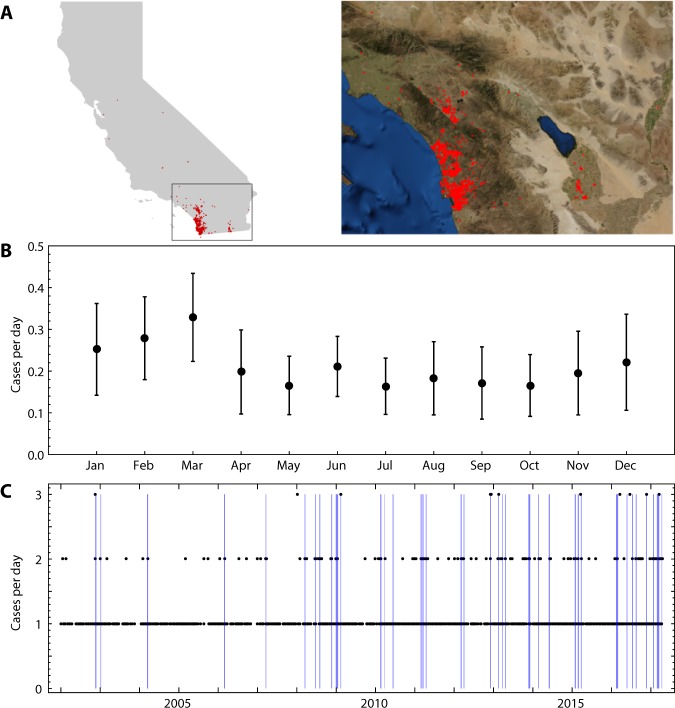
(**A**) Location of primary residence for KD cases treated at Rady Children’s Hospital San Diego, a regional pediatric medical center, between 1 January 2002 and 15 April 2017. Background imagery from the Wolfram Mathematica Knowledgebase^31^. (**B**) Average number of cases per day for each month of the year in the San Diego region. Error bars show the standard deviations over the sample period. (**C**) Number of cases for each day (black dots) over the study period; no points are plotted for zero cases. Blue vertical bars show temporal clusters, defined as 7-day windows including onsets of 4 or more KD cases (see Materials and Methods, Table 1).

The geographical distribution of KD cases based on the location of patient’s primary residence is shown in Fig. 1A. KD incidence in San Diego follows a seasonal cycle that is similar to Japan, and more broadly, all sampled locations in the Northern Hemisphere (Fig. 1B)^4,5^. The main peak in case numbers is in the period from January through March, with a smaller peak in June and a nadir in cases in September and October.

**Figure 2:**
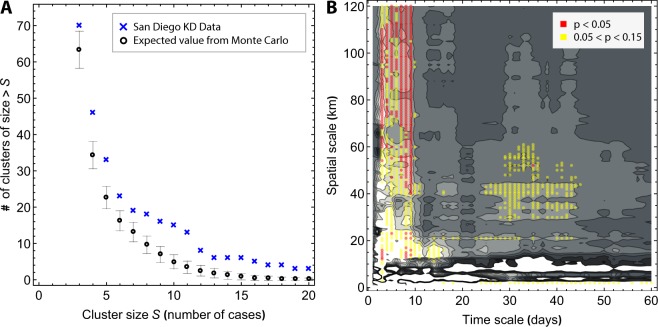
Robustness of temporal and spatiotemporal clustering of KD Cases in San Diego County. (**A**) The cluster size distribution of the construction shown in Fig. 1C. The number of clusters of with more than S cases are plotted against S. The result is compared with the corresponding curves obtained from a Monte Carlo simulation of synthetic time series that have the same average incidence and average seasonality as the San Diego KD time series, but for which there is no temporal structure apart from a regular seasonal pattern. The error bars are ±1 standard deviation. (**B**) Excess K statistic for spatiotemporal distribution of KD cases. Red indicates significant spatiotemporal clustering with *p* < 0.05, and yellow indicates 0.05 <*p*< 0.15.

In addition to the seasonal pattern of KD in San Diego, there are also anomalous high-incidence periods of shorter duration (Fig. 1C). Temporal clusters were defined as seven consecutive days encompassing the onset of four or more KD cases (see Materials and Methods for details of model construction). The first day of fever was defined as the onset of KD. We compared the cluster-size distribution with the range of cluster-size distributions generated by a Monte Carlo simulation under the null-hypothesis of no temporal structure apart from the seasonal pattern (Fig. 2A). The clusters were found to be statistically significant in that their size distribution could not be reproduced from a synthetic model. The same result was found for other cluster constructions that emphasized time scales of three days and 30 days, rather than the 7-day time scale. However, the spatiotemporal analysis discussed below revealed structures with durations of approximately one week, and consequently our further analyses focused on a temporal cluster definition of at least four cases within seven days.

### Spatiotemporal clustering

The temporal and spatial distributions of KD cases were not independent of each other. We used a modified Knox test^12,13^ similar to the one proposed by R. D. Baker^14^, to identify the number of pairs of KD cases that were close in both time and space, and computed the relative difference to the expected number of such pairs under the null hypothesis of no spatiotemporal clustering. This (excess) Knox statistic depends on the thresholds for what is considered to be close in time and space (Fig. 2B). Marked in red are the spatial and temporal scales for which the Knox statistic was significantly higher (p ≤ 0.05) than what was obtained from a Monte Carlo simulation for which the dependence between space and time was broken by shuffling the timing of the cases versus their locations. The results showed that KD clusters appeared at preferential time and space scales. This spatiotemporal clustering was most pronounced at time scales <10 days and spatial scales >10 km. These spatial scales are consistent with previous findings that there is no evidence of person-to-person transmission of KD^10,15^. (Additional information on spatial clustering can be found in the Supplementary Text and Figs S1 and S2).

### Association of temporal clusters and weather patterns

To investigate the relationship between weather patterns and KD incidence, we analyzed data of daily maximum and minimum temperatures, and precipitation, in the Southern California region. We also analyzed global atmospheric geopotential height fields (the 700 hPa geopotential height), whose temporal and spatial variability are closely related to that of the atmospheric pressure in the lower to middle troposphere and are generally indicative of the broad scale atmospheric circulation that dictates regional weather patterns. All data were de-seasonalized, and consistent anomaly associations were detected by constructing composite maps. These composites were based on a collection of dates flanking KD onsets and making comparisons with the expected distribution of such composites under the null hypothesis of no association between the chosen dates and particular anomalous weather and climate patterns. (See Materials and Methods, and Supplementary Information).

**Figure 3:**
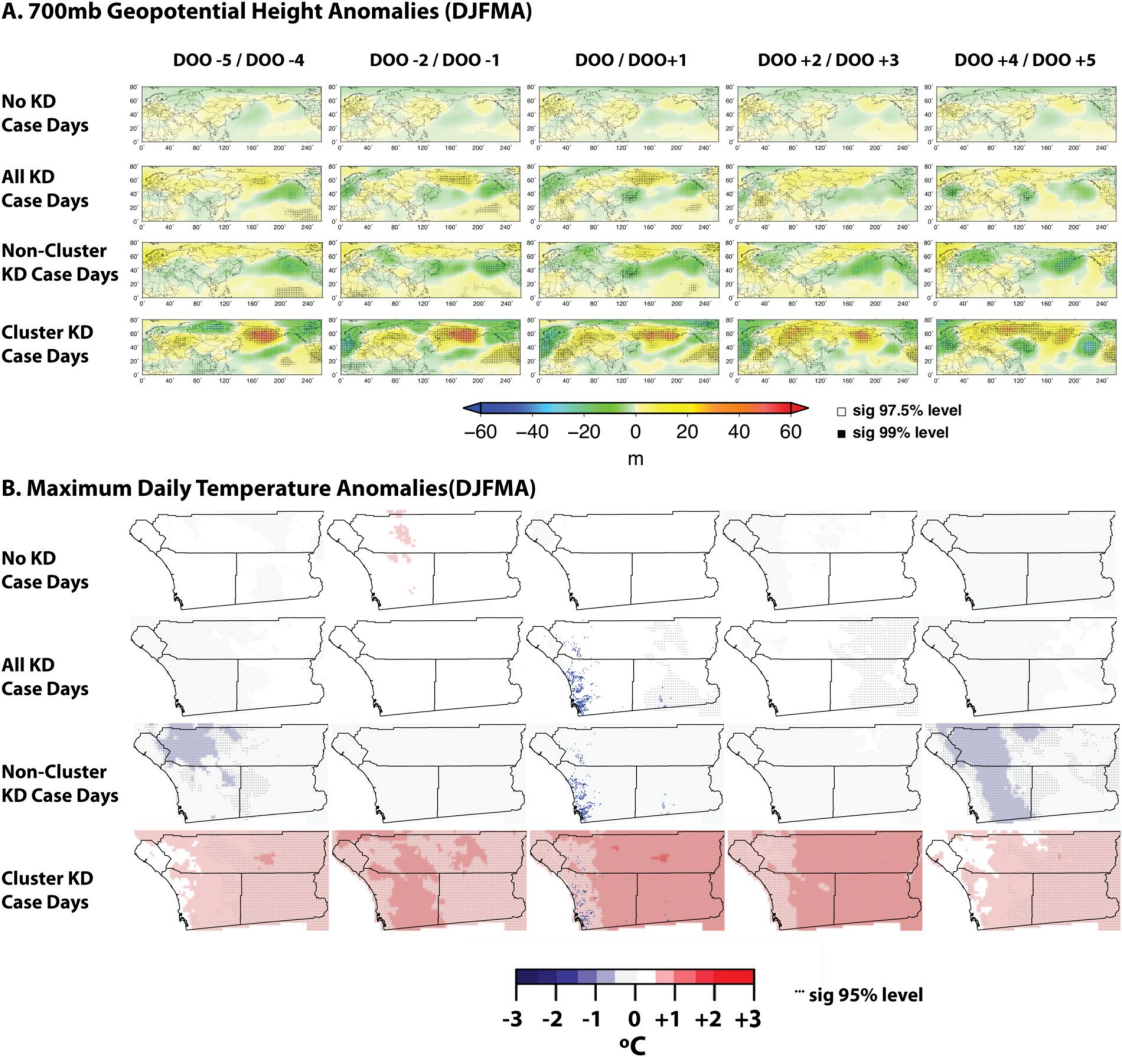
Composites of (**A**) 700 hPa geopotential height (700 mb height) and (**B**) daily maximum temperature for a sequence of days before and after each KD case within a given group of cases, from 4 and 5 days before (DOO - 5) to 4 and 5 days after (DOO + 5) date of onset (DOO). The groups of cases shown are: (2nd row from top of each panel) all KD cases; KD cases not falling into a temporal cluster (3rd row from top); and KD cases falling into a temporal cluster (bottom row). For comparison, composites associated with a random sampling of days having no KD cases is shown in the upper row in each panel. In both sets of figures, stippling indicates statistical significance of an anomaly composite in a given location. On the bottom (local) panel, the blue dots in the date of onset figure (center column) show where the KD cases occurred. Here we show only cases (and random, non-KD DOO equivalent days) from the main KD season, between December and April (DJFMA). Equivalent plots for other seasons and other variables (U-wind, V-wind, Minimum Daily Temperature, and Precipitation) are shown in the SI. The high pressure anomalies in the global figures for Cluster KD Case Days are consistent with reduced wind speeds across the Pacific and the high temperatures in the San Diego region.

We found that days with KD cases had slightly higher daytime temperatures and significantly warmer nighttime temperatures, and were also associated with excess rainfall north of San Diego in the main season (December-April, see Fig. 3 and Supplementary Fig. S7), but drier than average conditions in the secondary season (May-August, Supplementary Fig. S8). At the global scale, all KD case days taken together did not exhibit a strong geopotential height anomaly pattern, but there were some slight differences between the seasons (Supplementary Figs S3–S5).

The most significant heterogeneous signals emerged when cases were divided into those falling into the temporal clusters defined above (hereafter denoted as cluster cases) and those with date of onset outside of temporal clusters (non-cluster cases). There were strong temperature associations with temporal clusters. Cluster case days were warmer and drier than average, with slight differences between seasons (Fig. 3 and Supplementary Figs S6–S8). In addition, global circulation patterns (the 700 hPa geopotential height fields) were strikingly different between cluster and non-cluster case days (Fig. 3 and Supplementary Figs S3–S5). Non-cluster cases were associated with small (in magnitude) but significant negative anomalies across the North Pacific. In contrast, cluster case days were associated with an extensive swath of positive anomalies south of the Aleutian Islands and also, of direct relationship to weather in San Diego, a region of positive anomalies extending from the region offshore of Southern California eastward over the southwestern United States and northern Mexico. The unusually high 700 hPa anomalies are consistent with the warmer and drier than normal conditions exhibited by the regional temperature and precipitation composites described above. In both cases (cluster and non-cluster), these patterns build up over the period from 5 days before the KD onset date and dissipate, to some extent, by 5 days after onset. The patterns were especially strong in the secondary season (May-August, Supplementary Figs S5 and S8).

### Gene expression analysis and clinical presentations

**Figure 4:**
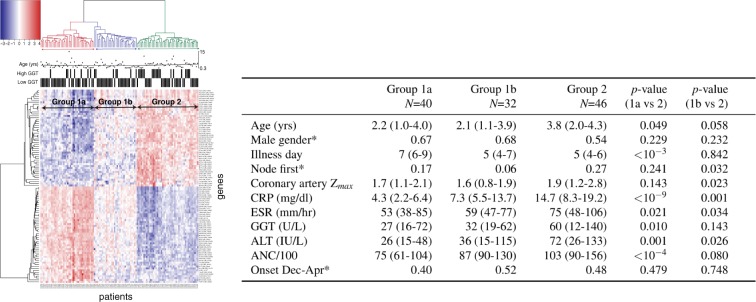
Unique grouping of gene expression patterns in KD patients. (Left) Heat map of the top 100 genes that were significantly differentially expressed between acute and convalescent phases in KD patients who were not in clusters, adjusted for illness day of acute blood sample collected on days 2–10 after fever onset. Rows represent 100 genes and columns represent 118 acute phase patient samples. Each cell value represents the intensity of mRNA expression in log-scale. Two patient phenotypes, age and GGT values, are displayed at the top. Annotation at top: Horizontal line is median age (2.8 yrs.), High GGT = > 3 SD above upper limit of normal for age reference range (Right). Demographic and clinical characteristics associated with 118 KD patients grouped based on unsupervised clustering of gene expression patterns. Clinical laboratory data were obtained before treatment with IVIG and at the same phlebotomy as the acute samples for gene expression. Values are median and IQR except those marked with *, which are ratios. Comparisons by Mann-Whitney test for continuous variables, and a *χ*^2^ test for proportions. (A larger version of the heat map is included in the Supplementary Information).

A previously published transcriptome database was available for a subset of 138 patients with gamma-glutamyl transpeptidase (GGT) values as a marker of oxidative stress included in this analysis^16^. Whole blood RNA was collected in the acute phase of the illness, prior to intravenous immunoglobulin (IVIG) administration, and again during the convalescent phase when inflammatory markers had returned to normal. Transcript abundance was estimated for 47,181 probes, and a volcano plot selected the top 100 probes that were most (with respect to the joint filtering criteria) differentially expressed between the acute and convalescent phases (Supplementary Fig. S9). Figure 4 shows a presentation of this gene expression analysis as a heat map with dendrograms based on unsupervised, hierarchical clustering with the average-linkage method using Euclidean distance (see Materials and Methods, Supplementary Information text, and Supplementary Figs S9–S11). To avoid confounding temporal clustering with gene expression analysis (and because representation in temporal clusters was small), we restricted this analysis to the 118 patients *not* in temporal clusters.

In the first level of the hierarchical clustering, the 118 KD patients were divided into three main groups (denoted group 1a, 1b, and 2) based on the transcript abundance patterns of the top 100 differentially expressed genes. (Fig. 4). Selected clinical characteristics of the patients in each of these groups were compared (Fig. 4, right panel). Compared to Group 2, Group 1a was characterized by younger age, later diagnosis (illness day), lower levels of inflammatory and oxidative stress markers, and reduced likelihood of presenting with fever and an enlarged cervical lymph node mass before the appearance of other clinical features (node-first presentation)^7^. Group 1b was similar in age to Group 1a and least likely to present with the lymph node-first pattern. The markers of inflammation for Group 1b were intermediate between Groups 1a and 2. The higher levels of CRP and ANC in Group 2 could be partially explained by earlier illness day at diagnosis (the interval between fever onset and RNA collection) despite adjustment for illness day in the heat map^17^. However, the higher levels of erythrocyte sedimentation rate (ESR), GGT, and alanine aminotransferase (ALT) are not known to be associated with earlier diagnosis or other demographic features.

### Temporal and spatial structure of clinical characteristics

**Figure 5:**
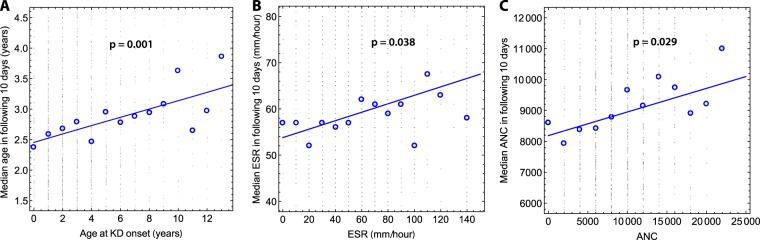
(**A**) Shows the median age in a 10-day window conditioned on the age of a reference patient with onset immediately prior to the beginning of the time interval. Fine black dots show all cases, blue circles show median values for a given bin. The p-value indicates the significance of a non-zero slope in the linear regression. (**B**) As in (**A**) but for erythrocyte sedimentation rate (ESR). (**C**) As in (**A**,**B**), but for absolute neutrophil count (ANC). R^2^ values for the three panels are 0.55, 0.29, and 0.47, respectively.

The gene expression analysis served to identify clinical variables that revealed a population substructure. Interestingly, we found no average difference in clinical presentation between patients in temporal clusters and those not in temporal clusters (Supplementary Table S1), but that patients with similar values for some of these variables nevertheless clustered in time. For example, we found that patient age, ESR and absolute neutrophil count (ANC) were autocorrelated in time: the median age of all KD patients with onsets within a 10-day interval following the onset of a reference patient was positively correlated to the age of the reference patient. This relationship was also observed for ESR and ANC values (Fig. 5). We also noted that the proportions of patients with elevated GGT values (defined as >3 times the age-adjusted reference range) clustered spatially (Supplementary Figs S1 and S2). The significance of non-random spatial distribution was tested through Monte Carlo simulations.

## Discussion

Defining a disease in space and time can generate hypotheses about its etiology. We report here that KD in San Diego County has both a seasonal structure, a shorter scale temporal clustering, and spatiotemporal clustering associated with temporal scales of up to 10 days and spatial scales of tens of kilometers. Temporal clusters were strongly associated with warmer and drier than average days and global circulation patterns with positive anomalies extending from the region offshore of Southern California eastward over the southwestern United States and northern Mexico. These unusually high 700 hPa anomalies were consistent with the warmer and drier than normal conditions exhibited by the regional temperature and precipitation composites. Patients clustered in time shared clinical characteristics including age and measures of inflammation. Finally, whole blood gene expression analysis suggested different patterns of host response consistent with either different environmental triggers or different intensities of exposure to the same agent. These observations are consistent with environmental triggers that occur in discrete outbursts associated with specific climate conditions over a limited geographic area that result in clusters of KD cases.

Spatial and spatiotemporal clustering of KD in San Diego was previously reported by Kao *et al*. using a much smaller dataset^18^. The larger dataset used in the current study extends these results in detecting spatiotemporal clusters that occupy a wider range of spatial and temporal scales. Temporal and geographic clustering was previously reported from Japan attributed to “unknown region-specific factors”, but no analysis of climate or environmental factors was performed^19^. These observations support previous findings^20^ that an environmental exposure, related to tropospheric wind patterns, is a trigger for KD. The anomalies in 700 hPa with high pressure off the Aleutian Islands and the Southern California coast might be expected to influence trans-Pacific circulation patterns and winds over San Diego, as shown in the U-wind and V-wind anomaly composites in Supplementary Figs S3–S5.

A coherent seasonality for KD has been established for the Northern Hemisphere^4^, which strongly suggests an environmental driver of the disease. However, it is not known whether there are direct environmental triggers or causal exposures, or if the seasonality is an indirect effect, for instance that KD is triggered by a seasonal infectious disease. Features of KD epidemiology are consistent with both hypotheses, and to date, no environmental trigger has been identified, nor has a relation to any infectious agent been established. Chang *et al*.^21^ found associations between KD and viral infections, but no single virus could explain the occurrence of KD in the study. These authors postulated that different viral infections can trigger KD in susceptible individuals, and that the differences in KD seasonality for different parts of the world indicate that different viral infections are dominant in different geographic regions. A recent study in Toronto suggested that an antecedent minor infectious process primed the immune system, which then over-reacted upon encountering the KD trigger^10^. Data were available for the Toronto region that further suggested an association between KD incidence and exposure to aerosols containing a greater density of fungal species.

The clustering results suggest that the trigger for KD is not only driven by the long-term average climate that characterizes a season, but also by specific patterns that influence conditions at shorter time scales. This conclusion is drawn from a consistent set of anomalous regional temperature and precipitation patterns and larger scale anomalous atmospheric motifs that occurred coincident with the KD clusters. Taken together, these findings paint a picture that is consistent with different environmental triggers for KD or differences in intensity of exposure to the same trigger. Gene expression analysis with unsupervised clustering was used to uncover patterns of host response that might be linked to the inciting environmental exposure. Surprisingly, the cohort organized itself into three main groups based on patterns of the top 100 differentially expressed genes. These distinct patterns were further associated with different clinical phenotypes, again supporting the concept of different environmental exposures. Although host genetics underlie susceptibility to KD, children with shared genetic background would not be expected to cluster in space. An exploration of clustering by ethnicities yielded no consistent pattern (data not shown). Our data raise the possibility of different genetic susceptibilities for different environmental triggers.

The spatial distribution of KD patients with elevated serum levels of GGT is intriguing and may be an important clue to the inciting agent. This cell surface enzyme that cleaves extracellular glutathione (G-SH) or other gamma-glutamyl compounds is found in many tissues throughout the body. GGT serves to increase the availability of amino acids, primarily cysteine, for intracellular G-SH synthesis and plays a crucial role in maintaining G-SH homeostasis and defense against oxidative stress^22^. Levels in the serum are elevated in conditions involving oxidative stress including infection and inflammation. GGT as a marker of host response to oxidative stress suggests that spatial clusters may be linked to locally released triggers that cause either more or less oxidative stress.

Our analyses have potentially broad implications for KD research. First, they suggest that attempts to define both the etiology of KD and the pattern of host genetics influencing susceptibility may have been confounded by the assumption that there was a single trigger affecting a genetically homogeneous population. The fact that clusters were comprised of either younger or older patients is consistent with the hypothesis that there may be different triggers for KD. It logically follows that one trigger may be common in the environment and thus exposure occurs early in life and the children with genetic susceptibility to this common trigger will tend to be younger. The second trigger may be rarer and thus children with genetic susceptibility to this second agent may not encounter it until they are older. Alternatively, the different groups could represent different genetic susceptibilities to the same agent with one group requiring a higher level of exposure that occurs less frequently and thus this group would be older. These interpretations would be consistent with recent observations from Japan that uncovered an association between age at onset and season with younger patients more likely to present with acute KD in the summer and fall months^23^.

We recognize several limitations to our study. First, the spatiotemporal analyses were performed in a single geographic region and validation in an independent site was not performed. Thus, our findings should be viewed as hypothesis-generating and Japan would be a logical region in which to test the hypothesis of spatiotemporal clustering and relationship to large scale climate patterns. Whole blood RNA samples were available for only a small subset of the study population, thus precluding an analysis of gene expression pattern by membership in a temporal cluster. As with all climate science, the analysis here relied on statistical inference and correlations and cannot be directly experimentally tested.

## Conclusion

Spatiotemporal clustering of KD associated with regional weather and climate anomalies suggests environmental triggers acting over a broad geographic area. The segregation of clinical phenotypes with distinct gene expression patterns further suggests that different exposures, either in nature or intensity, affect genetically distinct populations of children. Studies of host genetics and the triggers for KD should focus on patient clusters that share common demographic and clinical features.

## Methods

### Subjects

The Kawasaki Disease Research Center at University of California, San Diego/Rady Children’s Hospital San Diego (RCHSD) maintains a database of prospectively collected information on patients treated for KD at RCHSD, a free-standing children’s hospital that is the principal pediatric inpatient facility for a population of approximately 3.5 million. All patients diagnosed with KD between January 1st, 2002 and April 15th, 2017 in our database were included in the analysis. Prospectively collected data included age and date at onset, sex, self-reported race/ethnicity of both biologic parents, street address for geospatial mapping, laboratory and echocardiographic data, and additional clinical presentation details. The Institutional Review Board at UCSD approved the prospective collection of de-identified data, all research was performed in accordance with relevant guidelines and regulations, and written informed consent was obtained from the parents and assent from the patients as appropriate.

### Gene expression analysis

Details of the patient cohort and microarray data preprocessing steps have been previously published^16^. Briefly, whole blood RNA was collected in PAXgene tubes during the acute phase, prior to IVIG administration, and during the convalescent phase after the resolution of inflammation from 138 KD whose acute samples were obtained within the first 10 days after fever onset. Complete blood counts and other clinical laboratory testing were performed on the same blood sample used for transcript analysis. Coronary artery dimensions were described by the variable Z_*max*_, which was defined as the maximal Z score (standard deviation units from the mean) of the internal diameter of the left anterior descending and right coronary arteries normalized for body surface area during the first six weeks after illness onset. Transcript abundance was determined using the the Illumina HumanRef-12 V4 BeadChip containing probes for more than 47,000 gene transcripts. Quantile-quantile normalization was applied to mRNA expression values to remove between-sample variation originated from the non-biological source^24^. A linear model and empirical Bayes method were adopted to assess the differential expression between two phases, acute vs. convalescent, adjusted by illness day^25^. Top differentially expressed genes were selected by fold-change between two phases and False Discovery Rate (FDR) adjusted p-values to correct for multiple testing^26^. Hierarchical clustering analysis was done with different linkage methods using Euclidean distance on gene expression values after centering and scaling at sample level. After multiple clustering results were evaluated by Dunn index^27^ and silhouette index^28^, the final version was displayed in a heatmap. (Additional details are provided in the Supplementary Information).

### Temporal cluster construction

The temporal clusters shown in Fig. 1C were constructed using a two-stage definition. By using a 7-day moving window over the time period from January 2002 to April 2017, we first defined all of the days in the analysis period as “cluster” days if they fell within a 7-day window that contained 4 or more cases, or “non-cluster” days if they were not part of a 7-day window with at least 4 KD cases. Temporal Clusters were then defined as unbroken sequences of “cluster days” (the two shown below are 7 and 11 days, respectively), and “Cluster KD Cases” defined as the cases contained within those time periods.

### Monte Carlo Methods

To understand whether the patterns we observed were statistically different from what would occur by chance alone, we used a series of Monte-Carlo style reshuffling exercises. For the temporal clustering, we randomly distributed the same number of cases over the time period, with the observed seasonality, and counted the number of clusters using the method described above. We repeated this exercise 500 times to define the probability distribution of number clusters occurring by chance alone, and compared the p-value for the t-statistic of the observed number of clusters in the KD dataset versus the number of clusters in the simulated distribution.

For the spatio-temporal clustering, we similarly “shuffled” the observed data by randomly assigning the locations (GPS coordinates of the home address) of existing cases to the dates of onset of those cases. We then compared the degree of spatiotemporal clustering (excess Knox statistic) of the observed KD cases to the simulated distribution for which location and date of onset were by definition statistically independent.

### Analysis of climate patterns

To analyze weather associations with dates leading up to, and following, KD onset, we used two sources of data. For regional (southwestern United States) patterns, we used the PRISM data maintained by Oregon State University^29^. For three variables (Maximum Daily Temperature, Minimum Daily Temperature, and Daily Precipitation), we constructed a climatology of day-of-year average conditions using a 5-day running mean and all data from 2002–2016. We then constructed daily anomalies (anomaly = daily condition − climatology) for each day in the time window of KD clinical data. For global-scale weather patterns, we followed a similar methodology using the NCEP/NCAR Reanalysis^30^, which provides a comprehensive global atmospheric data set for climate monitoring and research using a global atmospheric circulation model which assimilates available observations every 6 hours from 1948 to the present. Using NCEP/NCAR daily 700 hPa fields, we created climatology of day-of-year conditions using a 7-day running mean for 2002–2016, and then similarly constructed daily anomalies. We examined 1000 hPa (approximately surface level) temperature and precipitation data from the reanalysis dataset to ensure that the local PRISM data agreed with the NCAR data. We then prepared similar 700 hPa geopotential height anomaly (a proxy for pressure field) and vector wind (U- and V- winds) anomaly data sets.

To analyze potential association of climate conditions (both regional and global) with KD, we prepared composites of the anomalies for both the global circulation (geopotential height and vector wind) and local conditions (temperatures and precipitation) for different groups of cases. For each of the groups listed below, we extracted the dates of onset (DOO) in three seasonal designations - all months, the main KD season in San Diego (DJFMA), and the secondary season in San Diego (MJJA) (see Fig. 3 and Supplementary Figs S3–S8). The groups are: (a) Days without KD cases, (b) Days with KD cases (all cases whether in temporal clusters or not), (c) Days with KD cases not in temporal clusters only, (d) Days with KD cases in temporal clusters only.

For each of these 12 sets of dates (4 groups and 3 seasons), we created composites of the local temperature and precipitation and global circulation anomalies for all variables. We then defined statistical significance thresholds for these anomalies by calculating the t-statistic of the mean value of the composite anomaly and constructing a confidence interval (e.g., 95%). The significance markers in Fig. 3 (Supplementary Figs S3–S8) thus represent the level of confidence that the displayed anomaly distribution does not contain average climatology (i.e., that it is outside the expected range for those days). The method of using anomalies relative to daily climatology allowed us to compare days across different seasons relative to their normal conditions and to examine patterns beyond seasonality. The partitioning of the year into seasons then allowed us to explore if these dynamics differed over the course of the year.

**Table 1:** An illustrative example of temporal cluster construction, with a simulated set of 10 KD case onsets (second row) over a period of 22 consecutive days.

Day #	1	2	3	4	5	6	7	8	9	10	11	12	13	14	15	16	17	18	19	20	21	22
# of KD Cases	1	2	1	0	0	0	0	0	1	0	0	0	0	0	2	1	1	1	0	0	0	0
Cluster Day?	1	1	1	1	1	1	1	0	0	0	1	1	1	1	1	1	1	1	1	1	1	0
Cluster KD Day?	C	C	C						NC						C	C	C	C				
Cluster Size:	4 cases										5 cases											

By our definitions, this would be coded as 2 temporal clusters (with 4 and 5 cases, respectively); 9 of the 10 cases here would be Cluster KD cases, and 1 (occurring on Day 9) would be a Non-Cluster KD case. The “Cluster Day” row indicates whether a given day in the time series falls within any 7-day window that contains at least 4 KD onsets. “Cluster KD Day” indicates a KD onset day defined as within a temporal cluster. These are the designations used in the construction of climate anomalies, and in all analyses grouped by temporal cluster membership (‘C’ indicate Cluster KD Days, and ‘NC’ indicate Non-Cluster KD Days). Although the initial definition is at least 4 KD case onsets in a period of 7 days, the final cluster length can extend beyond 7 days and final cluster size can extend beyond 4 cases, since a cluster is extended until a “break” occurs and there is a non-cluster day (e.g., Day 22). This is why the vertical axis of Fig. 2 refers to clusters greater than a given size.

### Accession codes

The gene expression data used in this study can be accessed from the Gene Expression Omnibus (GEO) database with a series number GSE63381 (https://www.ncbi.nlm.nih.gov/geo/query/acc.cgi?acc=GSE63881).

## Electronic supplementary material

Supplementary Information
