# Author Correction: Clustering and climate associations of Kawasaki Disease in San Diego County suggest environmental triggers

**DOI:** 10.1038/s41598-019-42137-6

**Published:** 2019-05-09

**Authors:** Martin Rypdal, Veronika Rypdal, Jennifer A. Burney, Daniel Cayan, Emelia Bainto, Shannon Skochko, Adriana H. Tremoulet, Jessie Creamean, Chisato Shimizu, Jihoon Kim, Jane C. Burns

**Affiliations:** 1 Department of Mathematics and Statistics, UiT the Arctic University of Norway, Tromsø, 9037 Norway; 2 Department of Pediatrics, University Hospital of North Norway, and Department of Clinical Medicine, UiT the Arctic, University of Norway, Tromsø, 9037 Norway; 3 School of Global Policy and Strategy, University of California San Diego, La Jolla, CA 92093 USA; 4 Scripps Institution of Oceanography, University of California San Diego, La Jolla, CA 92093 USA; 5 Department of Pediatrics, University of California San Diego, La Jolla, CA 92093 USA; 6 Cooperative Institute for Research in Environmental Sciences, University of Colorado, Boulder, CO 80309 USA; 7 Physical Sciences Division, National Oceanic and Atmospheric Administration, Boulder, CO 80305 USA; 8 Department of Biomedical Informatics, University of California San Diego, La Jolla, CA 92093 USA

Correction to: *Scientific Reports* 10.1038/s41598-018-33124-4, published online 12 November 2018

Part of the paper presents results (including climate analysis) for temporal “clusters,” or time periods associated with anomalously high incidence rates of Kawasaki disease in the San Diego region. In the Article, these clusters were defined using a threshold density of at least 4 Kawasaki disease cases in a 7-day period. However, the analysis was conducted using a definition of 5 or more cases in a 7-day period. As a result, this Article contains errors.

The legend of Figure 1 is incorrect,

“Blue vertical bars show temporal clusters, defined as 7-day windows including onsets of 4 or more KD cases (see Materials and Methods, Table 1).”

should read:

“Blue vertical bars show temporal clusters, defined as 7-day windows including onsets of 5 or more KD cases (see Materials and Methods, Table 1).”

In the Results section, under the subheading ‘Seasonality and temporal clustering’,

“In addition to the seasonal pattern of KD in San Diego, there are also anomalous high-incidence periods of shorter duration (Fig. 1C). Temporal clusters were defined as seven consecutive days encompassing the onset of four or more KD cases (see Materials and Methods for details of model construction).”

should read:

“In addition to the seasonal pattern of KD in San Diego, there are also anomalous high-incidence periods of shorter duration (Fig. 1C). Temporal clusters were defined as seven consecutive days encompassing the onset of five or more KD cases (see Materials and Methods for details of model construction).”

Figure 2A should show the analysis corresponding to the temporal cluster construction that is used throughout the paper. The correct Figure 2A appears below.

**Figure 2:**
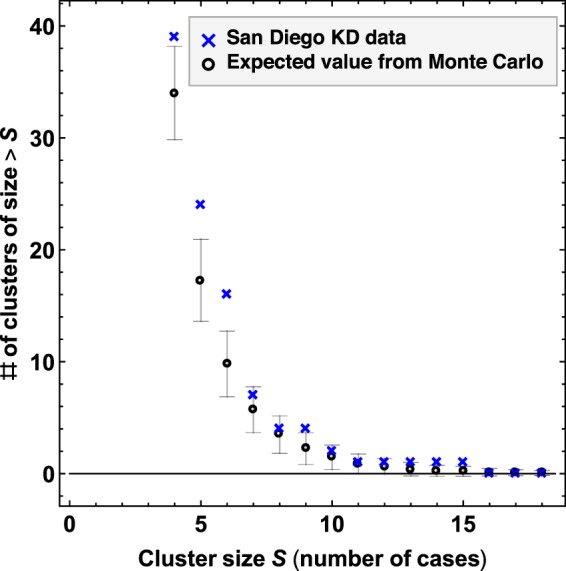
Robustness of temporal and spatiotemporal clustering of KD Cases in San Diego County. (**A**) The cluster size distribution of the construction shown in Fig. 1C. The number of clusters of with more than S cases are plotted against S. The result is compared with the corresponding curves obtained from a Monte Carlo simulation of synthetic time series that have the same average incidence and average seasonality as the San Diego KD time series, but for which there is no temporal structure apart from a regular seasonal pattern. The error bars are ±1 standard deviation. (**B**) Excess K statistic for spatiotemporal distribution of KD cases. Red indicates significant spatiotemporal clustering with *p* < 0.05, and yellow indicates 0.05 < *p* < 0.15.

In the Methods section, under the subheading ‘Temporal cluster construction’,

“By using a 7-day moving window over the time period from January 2002 to April 2017, we first defined all of the days in the analysis period as “cluster” days if they fell within a 7-day window that contained 4 or more cases, or “non-cluster” days if they were not part of a 7-day window with at least 4 KD cases.”

should read:

“By using a 7-day moving window over the time period from January 2002 to April 2017, we first defined all of the days in the analysis period as “cluster” days if they fell within a 7-day window that contained 5 or more cases, or “non-cluster” days if they were not part of a 7-day window with at least 5 KD cases.”

In the Methods section, under the subheading ‘Monte Carlo Methods’,

“For the temporal clustering, we randomly distributed the same number of cases over the time period, with the observed seasonality, and counted the number of clusters using the method described above.”

should read:

“For the temporal clustering, we randomly distributed the same number of cases over the time period, with the observed seasonality and a linear trend, and counted the number of clusters using the method described above.”

Finally, Table 1 should correspond to the temporal cluster construction (at least 5 cases in 7 days) that is used throughout the paper. As a result, the legend,

“An illustrative example of temporal cluster construction, with a simulated set of 10 KD case onsets (second row) over a period of 22 consecutive days. By our definitions, this would be coded as 2 temporal clusters (with 4 and 5 cases, respectively); 9 of the 10 cases here would be Cluster KD cases, and 1 (occurring on Day 9) would be a Non-Cluster KD case. The “Cluster Day” row indicates whether a given day in the time series falls within any 7-day window that contains at least 4 KD onsets. “Cluster KD Day” indicates a KD onset day defined as within a temporal cluster. These are the designations used in the construction of climate anomalies, and in all analyses grouped by temporal cluster membership (‘C’ indicate Cluster KD Days, and ‘NC’ indicate Non-Cluster KD Days). Although the initial definition is at least 4 KD case onsets in a period of 7 days, the final cluster length can extend beyond 7 days and final cluster size can extend beyond 4 cases, since a cluster is extended until a “break” occurs and there is a non-cluster day (e.g., Day 22). This is why the vertical axis of Fig. 2 refers to clusters greater than a given size.”

should read:

“An illustrative example of temporal cluster construction, with a simulated set of 11 KD case onsets (second row) over a period of 25 consecutive days. By our definitions, this would be coded as 2 temporal clusters (with 5 and 6 cases, respectively); 10 of the 11 cases here would be Cluster KD cases, and 2 (occurring on Days 9 and 25) would be a Non-Cluster KD cases. The “Cluster Day” row indicates whether a given day in the time series falls within any 7-day window that contains at least 4 KD onsets. “Cluster KD Day” indicates a KD onset day defined as within a temporal cluster. These are the designations used in the construction of climate anomalies, and in all analyses grouped by temporal cluster membership (‘C’ indicate Cluster KD Days, and ‘NC’ indicate Non-Cluster KD Days). Although the initial definition is at least 5 KD case onsets in a period of 7 days, the final cluster length can extend beyond 7 days and final cluster size can extend beyond 5 cases, since a cluster is extended until a “break” occurs and there is a non-cluster day (e.g., Day 22). This is why the vertical axis of Fig. 2 refers to clusters greater than a given size.”

The correct Table 1 and its accompanying legend appear below.

**Table 1:** An illustrative example of temporal cluster construction, with a simulated set of 11 KD case onsets (second row) over a period of 25 consecutive days.

Day #	1	2	3	4	5	6	7	8	9	10	11	12	13	14	15	16	17	18	19	20	21	22	23	24	25
# of KD Cases	2	2	1	0	0	0	0	0	1	0	0	0	0	0	2	1	1	1	1	0	0	0	0	0	1
Cluster Day?	1	1	1	1	1	1	1	0	0	0	0	1	1	1	1	1	1	1	1	1	1	0	0	0	0
Cluster KD Day?	C	C	C						NC						C	C	C	C	C						NC
Cluster Size	5 cases											6 cases													

By our definitions, this would be coded as 2 temporal clusters (with 5 and 6 cases, respectively); 10 of the 11 cases here would be Cluster KD cases, and 2 (occurring on Days 9 and 25) would be a Non-Cluster KD cases. The “Cluster Day” row indicates whether a given day in the time series falls within any 7-day window that contains at least 4 KD onsets. “Cluster KD Day” indicates a KD onset day defined as within a temporal cluster. These are the designations used in the construction of climate anomalies, and in all analyses grouped by temporal cluster membership (‘C’ indicate Cluster KD Days, and ‘NC’ indicate Non-Cluster KD Days). Although the initial definition is at least 5 KD case onsets in a period of 7 days, the final cluster length can extend beyond 7 days and final cluster size can extend beyond 5 cases, since a cluster is extended until a “break” occurs and there is a non-cluster day (e.g., Day 22). This is why the vertical axis of Fig. 2 refers to clusters greater than a given size.

