# Supplementary material for: Clustering and climate associations of Kawasaki Disease in San Diego County suggest environmental triggers
Source: Sci Rep. 2018 Nov 12;8:16140. doi: 10.1038/s41598-018-33124-4 (PMC6232126; doi:10.1038/s41598-018-33124-4)
Supplement: Supplementary file 1 — Supplementary Information [file 41598_2018_33124_MOESM1_ESM.pdf]

Supplementary Information for:

Clustering and climate associations of Kawasaki Disease in San Diego County suggest environmental triggers

Martin Rypdal, Veronika Rypdal, Jennifer A. Burney, Daniel Cayan, Emelia Bainto, Shannon Skochko, Adriana H. Tremoulet, Jessie Creamean, Chisato Shimizu, Jihoon Kim, Jane C. Burns

Corresponding Author: Jane Burns.
E-mail: jcburns@ucsd.edu

This PDF file includes:

Supplementary text
Figs. S1 to S11
Table S1
References for SI reference citations

Supporting Information Text

Spatial clustering

In addition to testing temporal and spatiotemporal clustering, we also tested for the presence of spatial clustering. The spatial clusters in Figures S1 and S2 were constructed using the agglomerate (hierarchical) clustering algorithm implemented in the Wolfram Mathematica software package. The purpose of these clusters was to establish predefined geographic regions to assess spatial variability in the proportion of patients with elevated GGT. The variance of the ratios between the geographic regions was compared with variances computed in a Monte Carlo simulation, and the null hypothesis (no spatial structure to the cases with elevated GGT within the general KD cohort) was rejected.

Gene expression clustering and evaluation

Hierarchical clustering was performed using Euclidean distance between two patients after centering by mean and scaling by standard deviation at sample level. Let C_i and C_j be two clusters and linkage methods to merge two clusters are defined as follows:

Average:
$$d_{complete}(C_i, C_j) = \frac{1}{|C_i||C_j|} \sum_{x \in C_i, y \in C_j} dist(x, y)$$

Centroid:
$$d_{centroid} = dist(\overline{x}, \overline{y})$$

Complete:
$$d_{complete}(C_i, C_j) = max_{x \in C_i, y \in C_j} dist(x, y)$$

Single:
$$d_{single}(C_i, C_j) = min_{x \in C_i, y \in C_j} dist(x, y)$$

Ward method:
$$d_{ward}(C_i, C_j) = \sum_{x \in (C_i \cup C_j)} d(x, m(C_i \cup C_j)) - \sum_{x \in C_i} d(x, m(C_i)) - \sum_{x \in C_j} d(x, m(C_j))$$

where $m(Y)$ is the center of the cluster Y.

The evaluation of clustering results were performed with four different methods as followings. The ratio between the average distance within clusters and the average distance between clusters. The lower ratio value represents the better clustering results. Dunn index is defined as the minimum average dissimilarity between two cluster divided by the maximum average within cluster dissimilarity. Normalized gamma is the correlation between distances and a 0-1-vector where 0 means same cluster, 1 means different clusters. Average silhouette width measures within-cluster similarity compared to the degree of between-cluster separation. The silhouette width for data i, $s(i)$, is defined as $s(i) = \frac{b(i)-a(i)}{max(a(i),b(i))}$ where $a(i)$ is the average distance between data i and all other data within the same cluster and $b(i)$ is the lowest average distance of i to all points in any other cluster The higher value of Dunn, gamma and silhouette with represents the better clustering results. Results from these tests are shown in Fig. S11

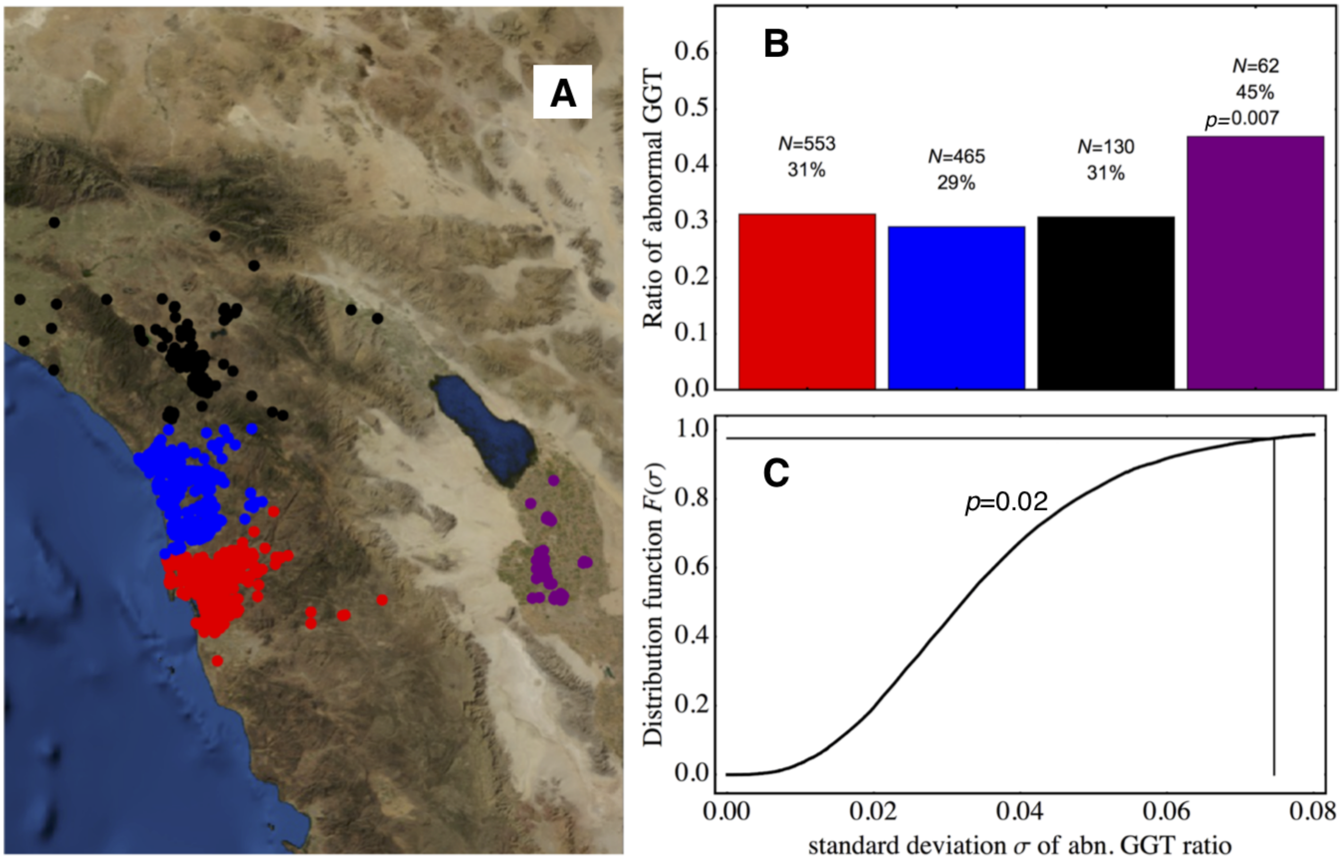

Fig. S1. Spatial clustering of KD in the central San Diego region. (A) Shows 4 geographical regions defined using an agglomerate method on the latitude-longitude coordinates for the onset locations. Background imagery from the Wolfram Mathematica Knowledgebase.(1) (B) Shows the ratio of elevated GGT in each of the regions shown in (A). The p-value associated with the eastern region is computed using a Monte-Carlo simulation where in each realization, 62 cases were drawn randomly from the cohort. (C) The distribution function for the standard deviation of the four ratio numbers shown in (B). The distribution is obtained from a Monte Carlo simulation where in each realization, 553, 485, 130 and 62 cases are randomly chosen from the cohort, and the ratios of elevated GGT are computed in each of the synthetic groups. The observed standard deviation, i.e. the standard deviations of the ratios plotted in (B), is shown as the vertical line, and the corresponding p-value in indicated in the plot.

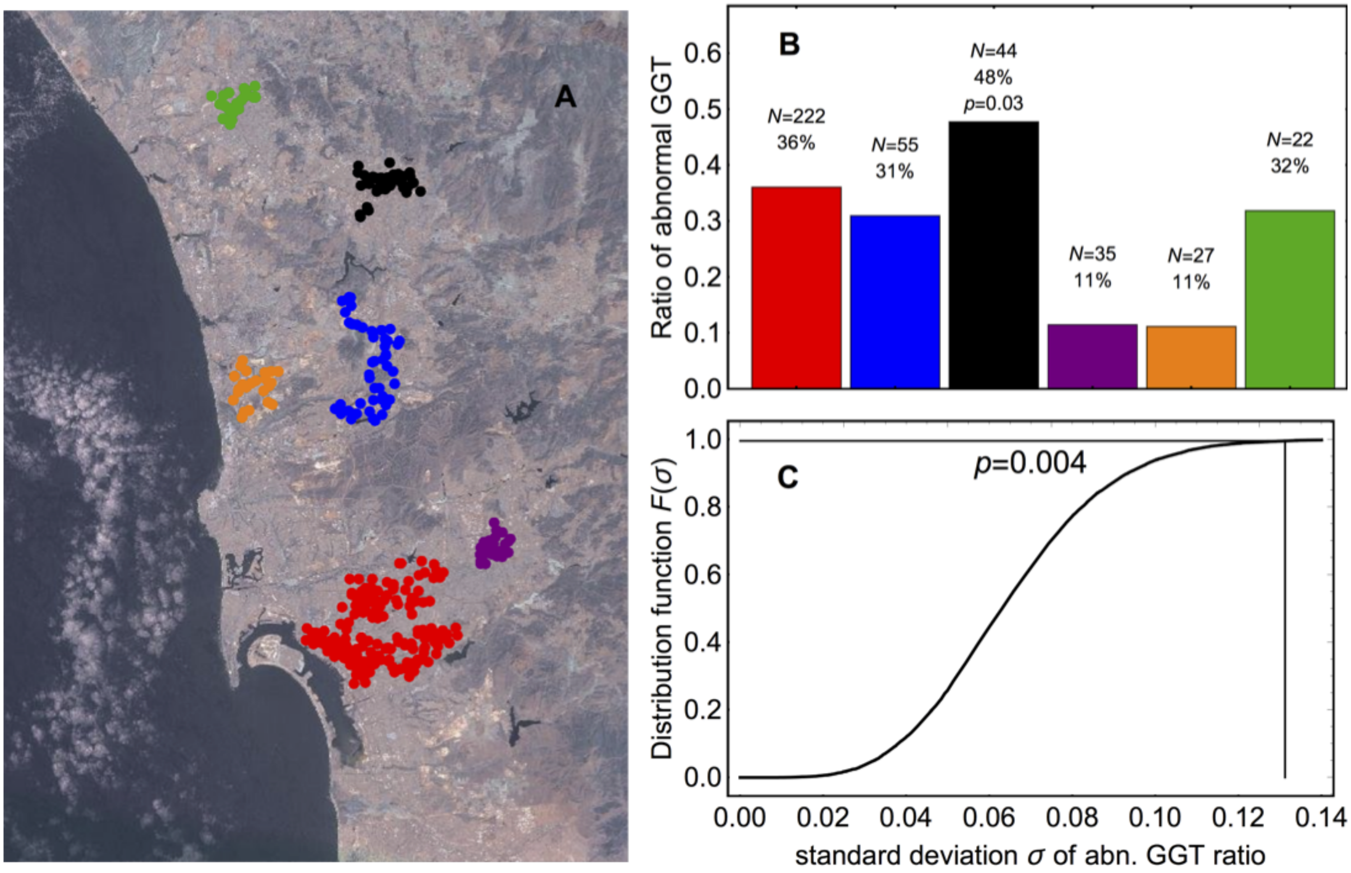

Fig. S2. As in Fig. S1, but with 6 areas in the coastal San Diego region. Background imagery from the Wolfram Mathematica Knowledgebase.(1)

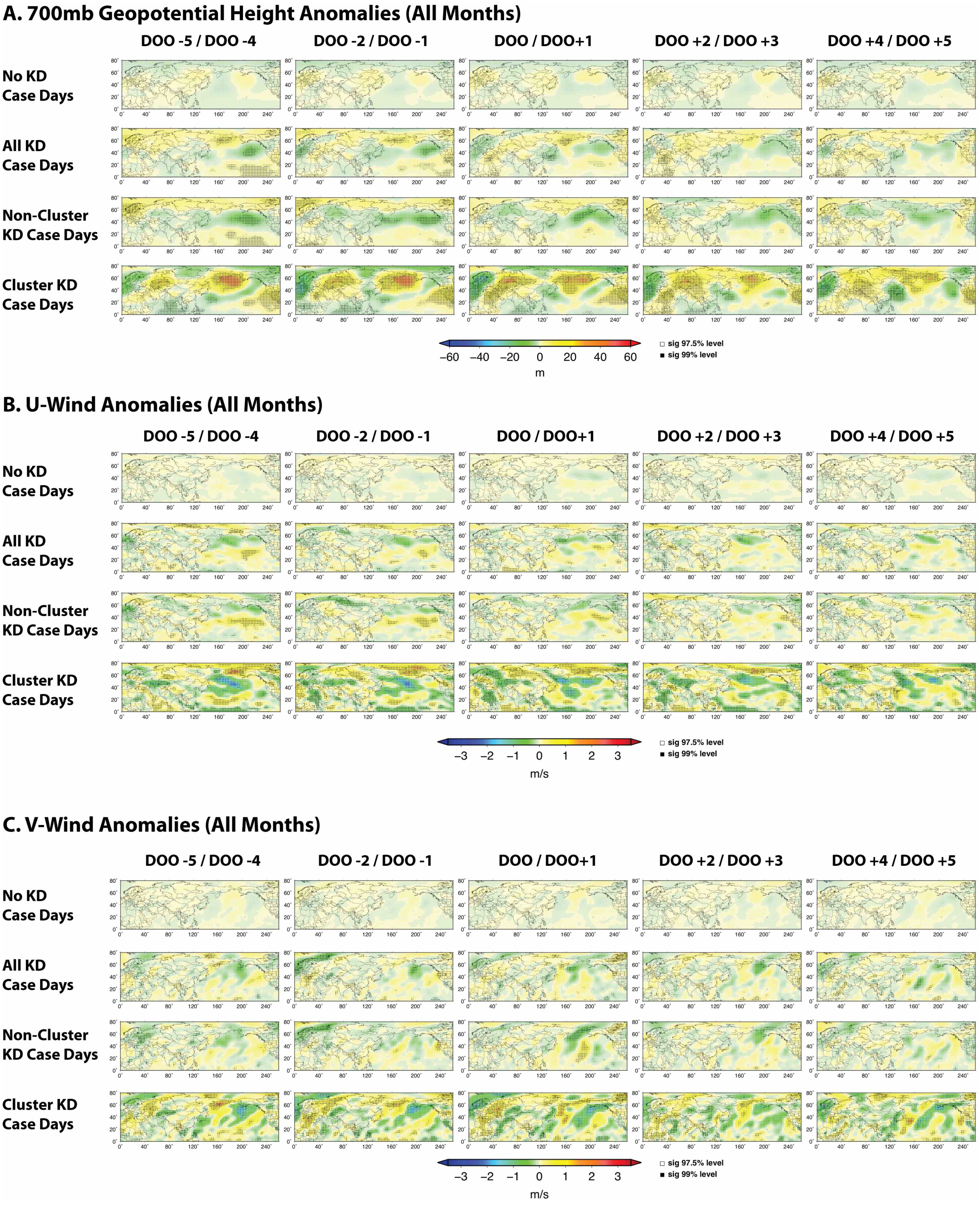

Fig. S3. Global (A) 700hPa (700mb) Geopotential height, (B) U-Wind, and (C) V-wind anomaly composites for four groups of days, from top to bottom in each panel: (i) an equivalent comparison group of days with no KD, (ii) all KD case days, (iii) KD cases not in temporal clusters, and (iv) KD cases in temporal clusters. Composites constructed leading up to date of onset (DOO) and after. Cases from the entire year.

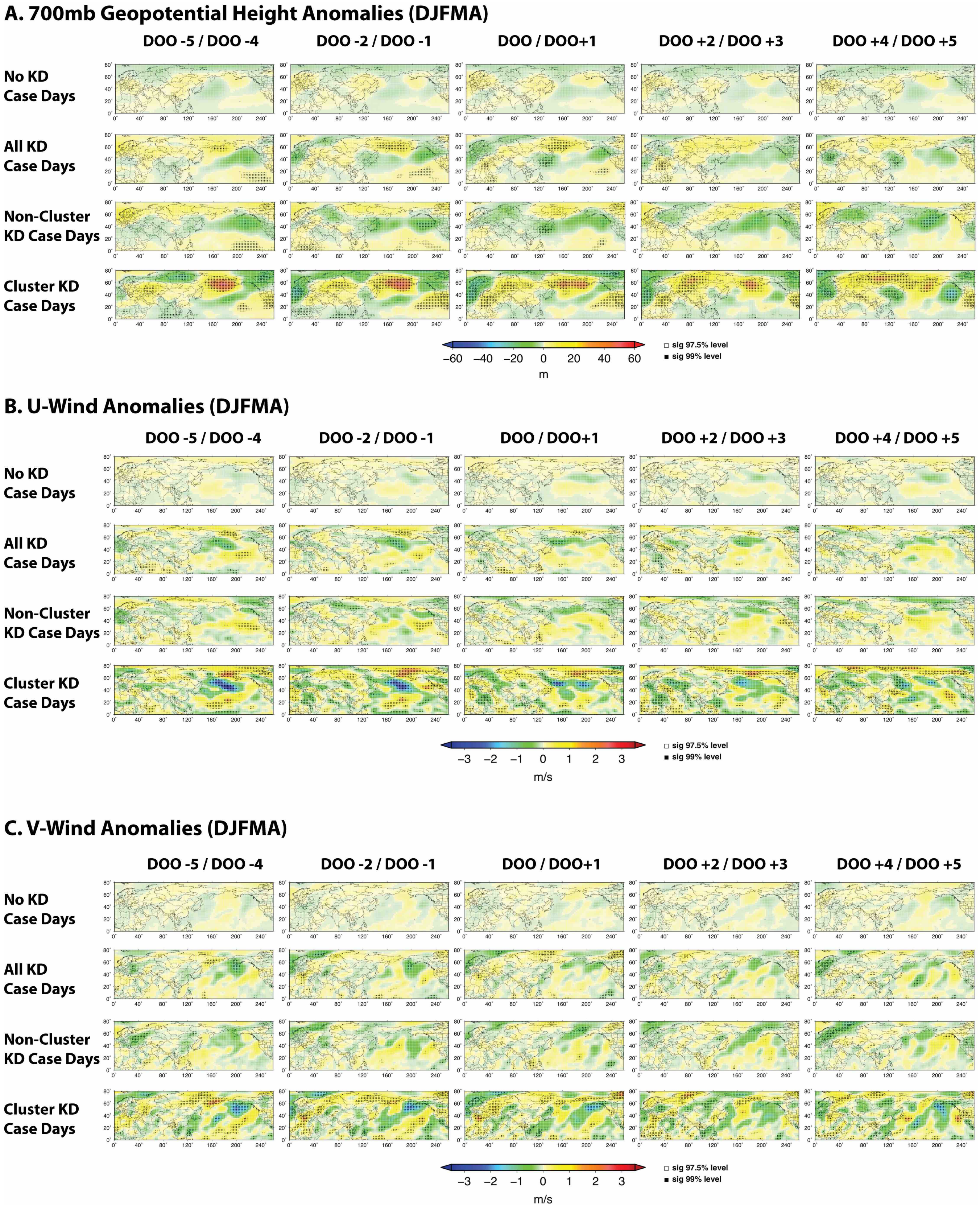

Fig. S4. As in Fig. S3 except that cases are drawn only from the main KD season in San Diego County, December-April.

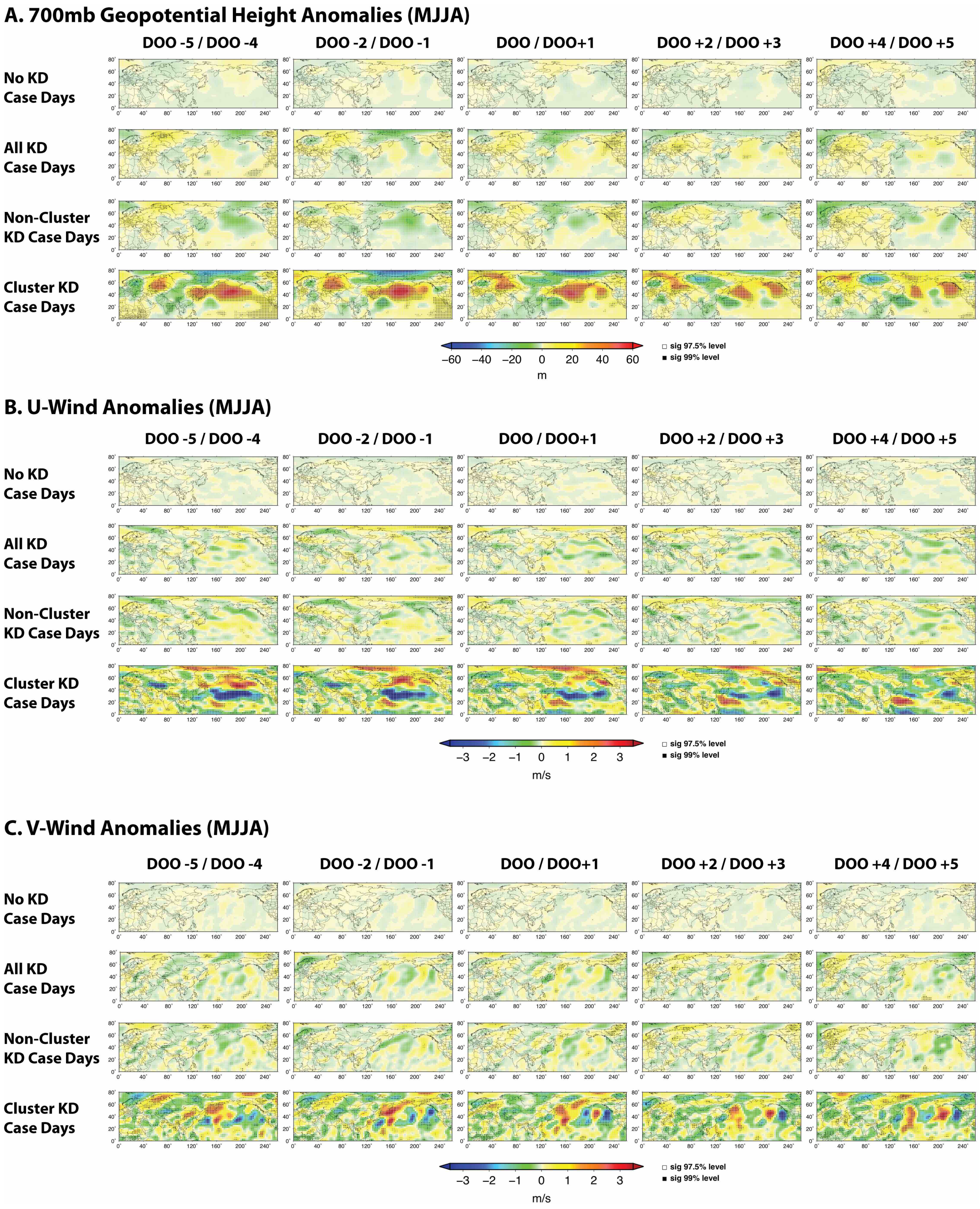

Fig. S5. As in Fig. S3 except that cases are drawn only from the secondary KD season in San Diego County, May-August.

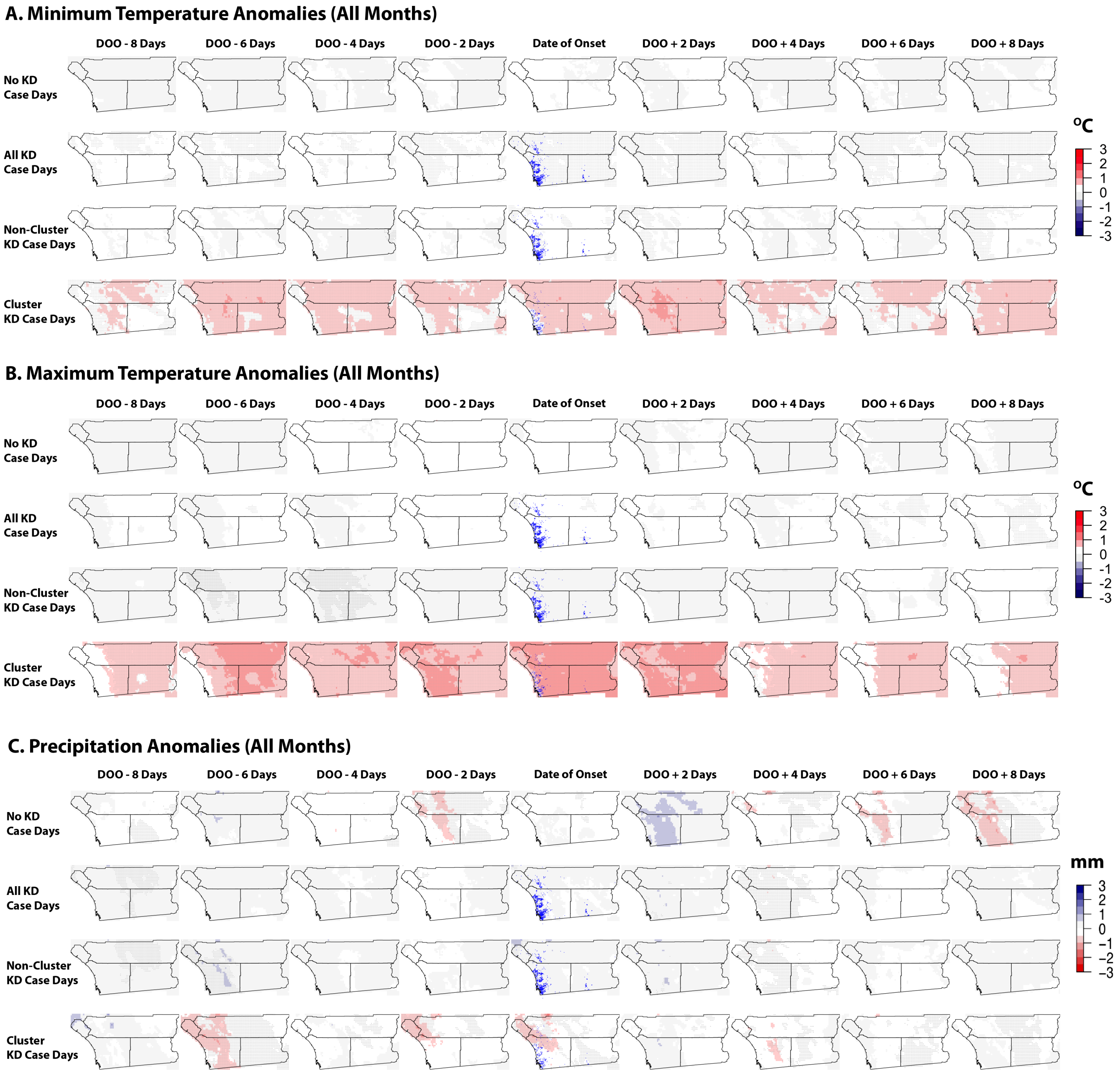

Fig. S6. Regional (Southern California) (A) Minimum Daily Temperature, (B) Maximum Daily Temperature, and (C) Precipitation anomaly composites for four groups of days, from top to bottom in each panel: (i) an equivalent comparison group of days with no KD, (ii) all KD case days, (iii) KD cases not in temporal clusters, and (iv) KD cases in temporal clusters. Composites constructed leading up to date of onset (DOO) and after. Cases from the entire year.

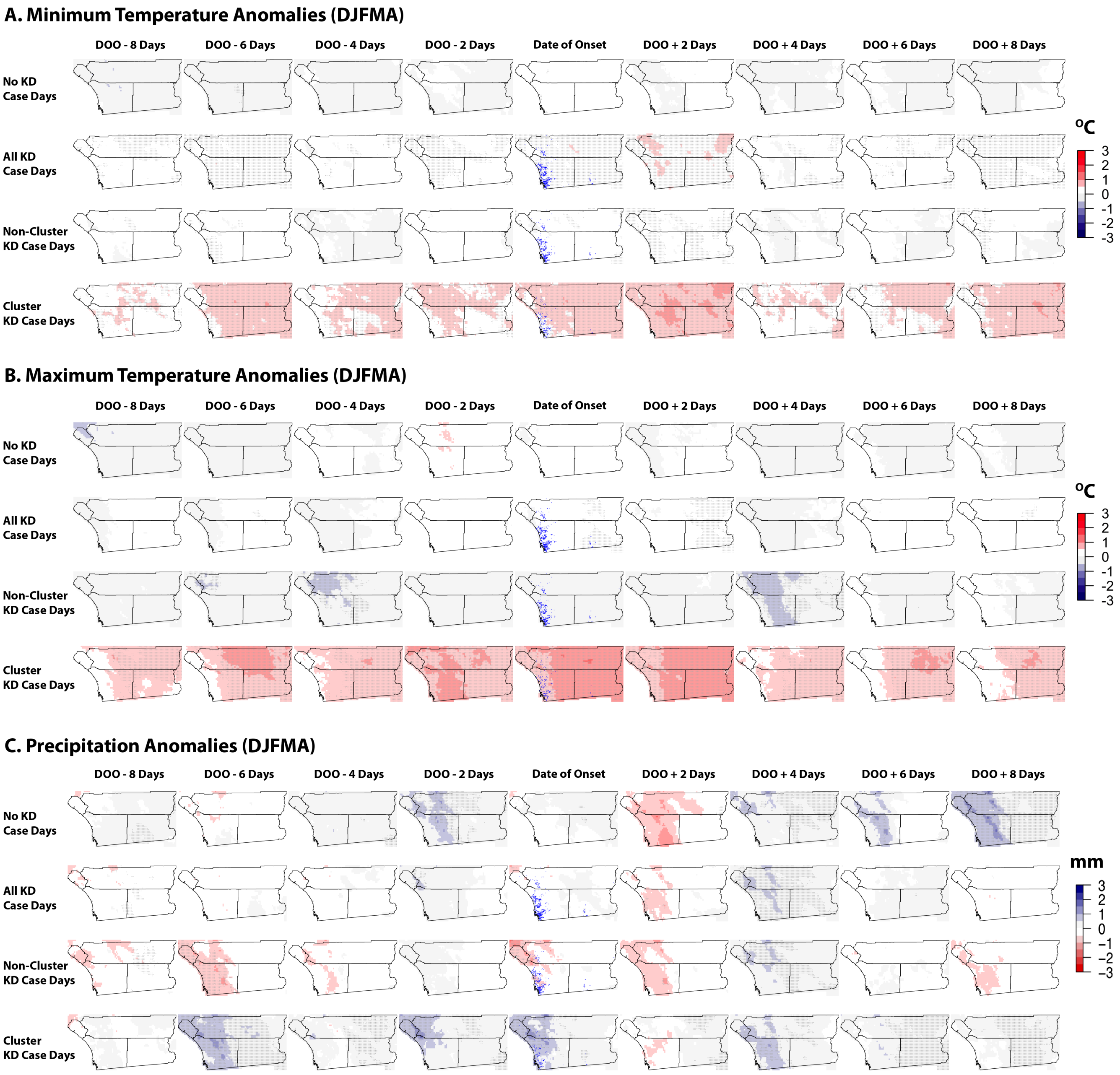

Fig. S7. As in Fig. S6 except that cases are drawn only from the main KD season in San Diego County, December-April.

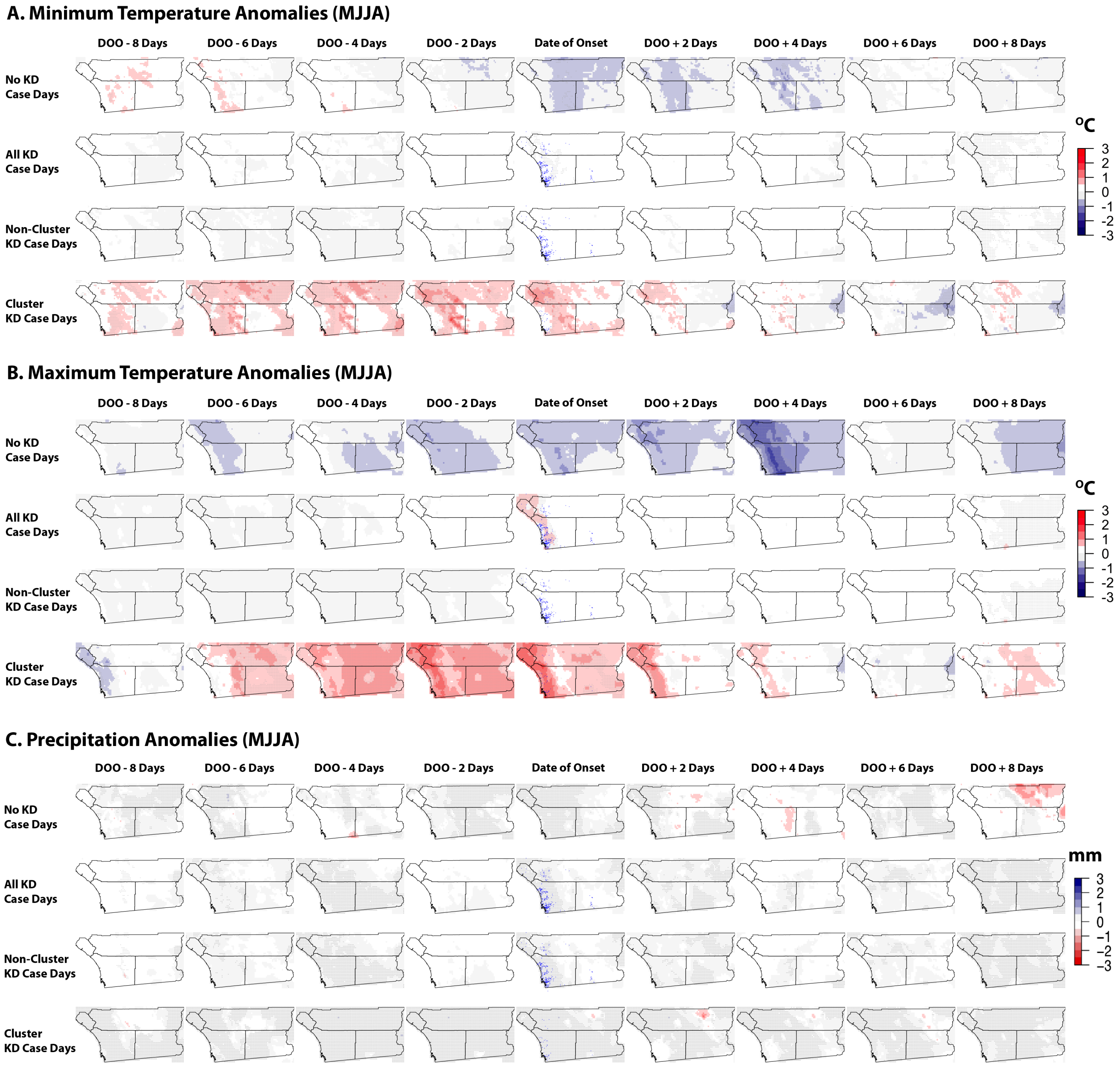

Fig. S8. As in Fig. S6 except that cases are drawn only from the secondary KD season in San Diego County, May-August.

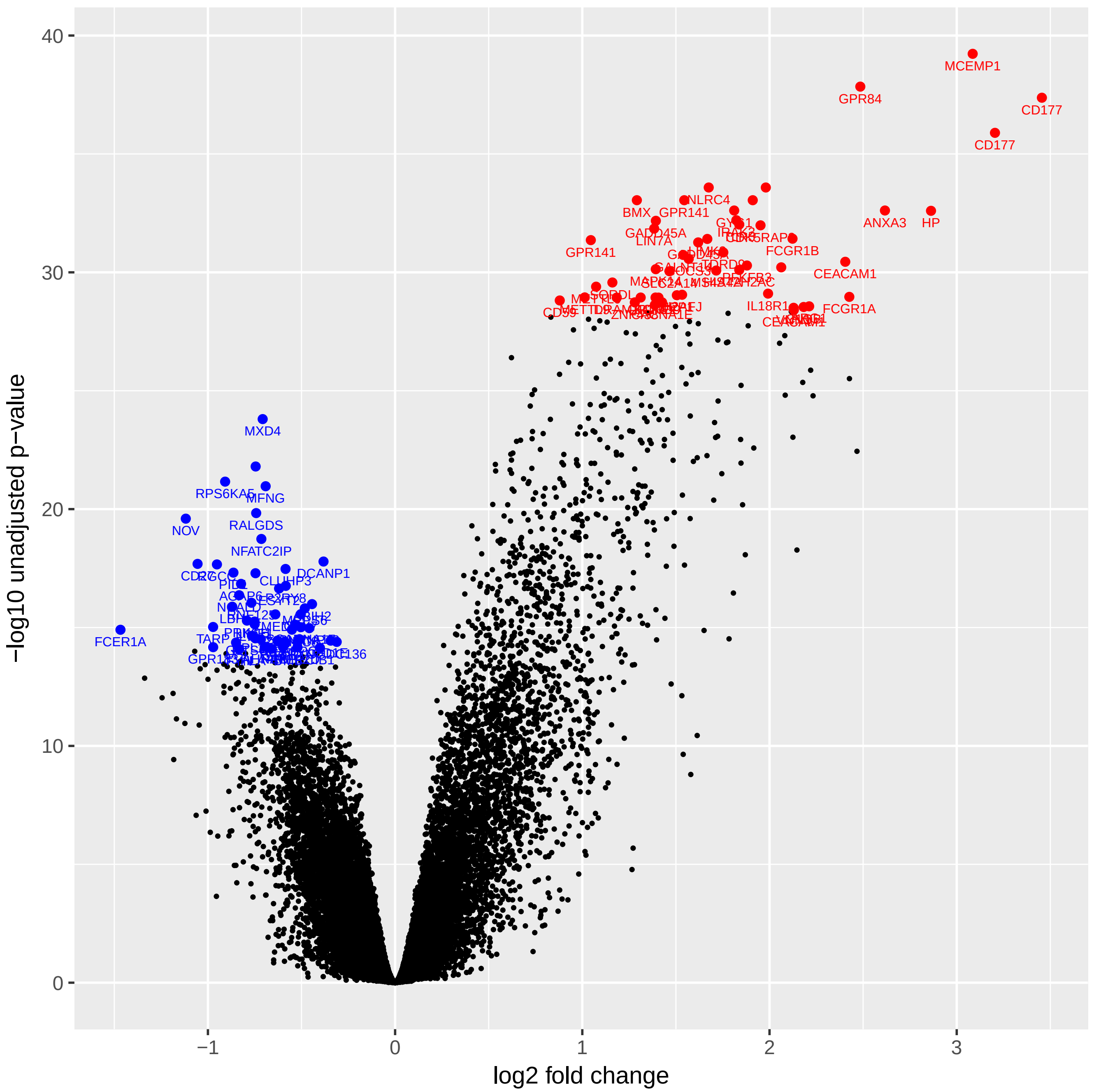

Fig. S9. Volcano plot. Y-axis represents the statistical significance of the fold-change in the form of unadjusted p-value from the test for phase effect (acute vs. convalescent) on gene expression adjusted by illness day. X-axis represents the gene expression signal in fold-change, a ratio between acute and convalescent phases. Top 50 genes whose fold change is greater than 1.5 and highly expressed in acute over convalescent conditions are selected by unadjusted p-value in ascending order and are colored in red, labeled with gene symbols. Top 50 genes highly expressed in convalescent over acute conditions are colored in blue.

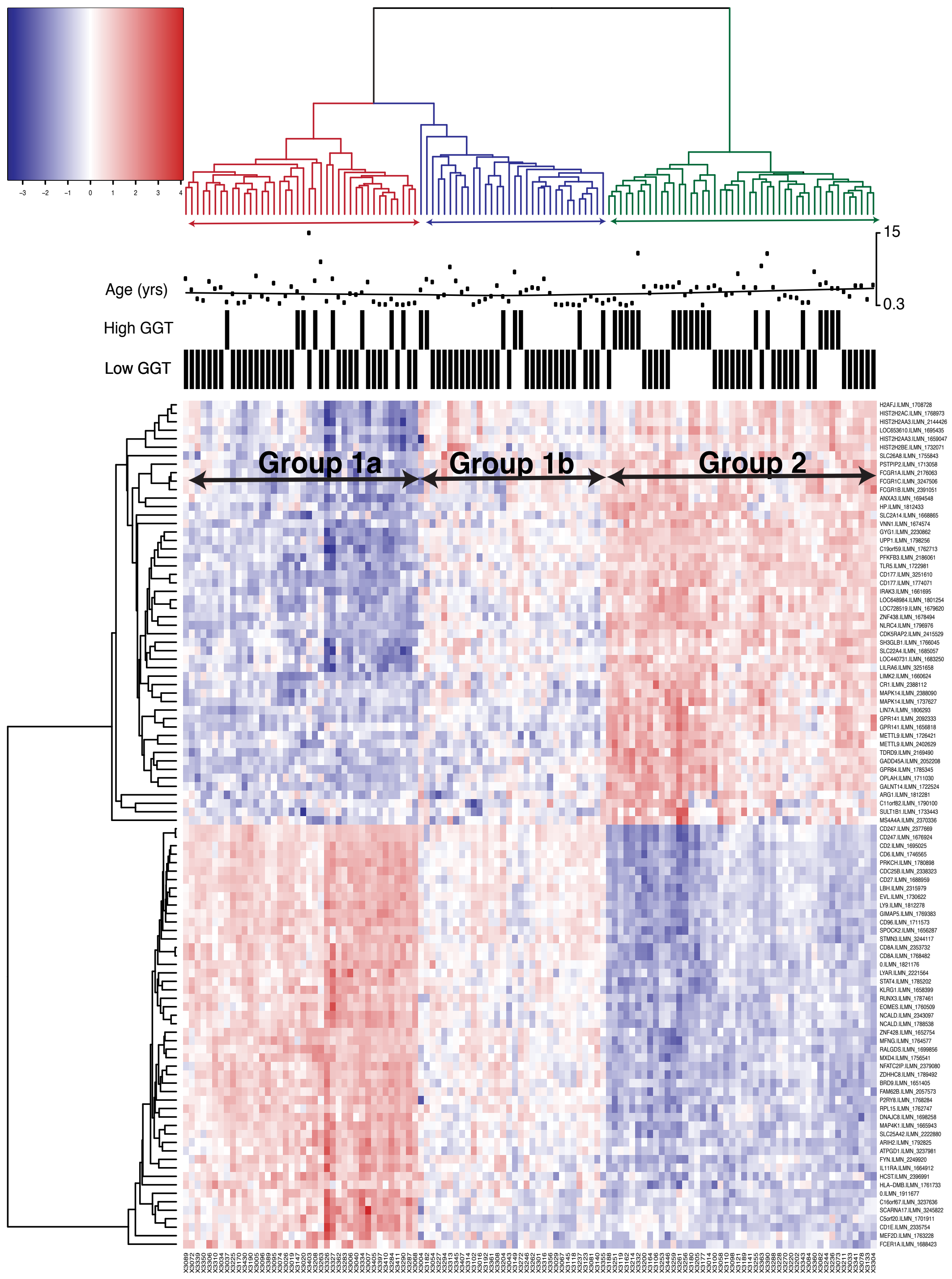

Fig. S10. Larger version of gene expression heat map shown in the main manuscript, Fig. 5. Heat map of the top 100 genes that were significantly differentially expressed between acute and convalescent phases in Kawasaki Disease patients, adjusted for illness day of acute blood sample collected on days 2-10 after fever onset. Rows represent 100 genes and columns represent 118 acute phase patient samples. Each cell value represents the intensity of mRNA expression in log-scale. Two patient phenotypes, age and GGT values, are displayed at the top. Gene expression in KD shows strong clustering.

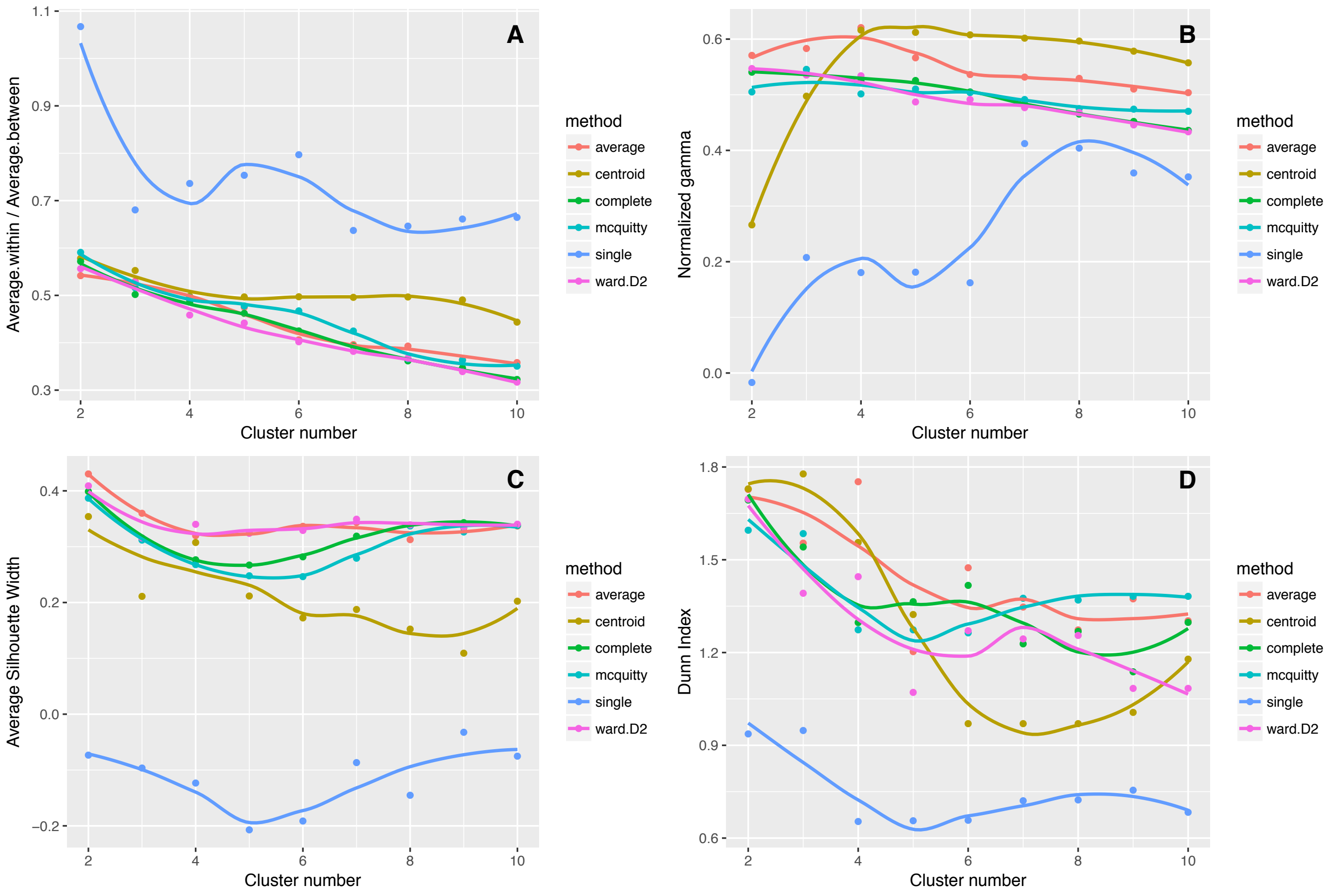

Fig. S11. Clustering result evaluation with four different metrics on y-axis along the different cluster number on the x-axis, varied by six different linkage methods to calculate the distance between two clusters as a function of the pairwise distances between cluster members. (A): The ratio of the average distance within clusters and the average distance between clusters (B): Normalized version of Pearson gamma that measures the correlation between distances and a 0-1 vector where 0 means same cluster, 1 means different clusters. (C): Average silhouette width that measures within-cluster similarity compared to the degree of between-cluster separation. (D): Dunn index defined as the minimum average dissimilarity between-cluster divided by the maximum average within-cluster dissimilarity.

Table S1. Although KD patient clinical characteristics are autocorrelated in time (Fig. 4, main manuscript), this not driven by temporal clusters. There are no average statistical differences between patients in temporal clusters and not in temporal clusters.

	Out of Cluster N=907	In Cluster N=257	p
Age (yrs)	2.6 (1.4-4.6)	2.9 (1.5-5.0)	NS
Male sex*	0.62	0.63	NS
Illness day	6 (5-8)	6 (5-8)	NS
Node first*	0.15	0.15	NS
Coronary artery Z-max	1.7 (1.1-2.6)	1.6 (1.0-2.3)	0.04^{α}
CRP (mg/dl)	6.6 (3.7-14.8)	6.0 (3.5-15.8)	NS
ESR (mm/hr)	58 (39-75)	59 (35-77)	NS
GGT (U/L)	40 (18-114)	42 (19-121)	NS
ALT (IU/L)	39 (22-104)	41 (24-109)	NS
ANC/100	85 (62-115)	88 (64-121)	NS
Onset Dec-Apr*	0.46	0.74	NS
Onset May-Nov*	0.54	0.26	NS

$^{\alpha}$: Skewed by three patients with z = 86, 37, 35 (very large aneurisms)

References

1. Wolfram Research Inc. (Accessed 30 May 2017) Mathematica, Version 11.1.1.0. Champaign, IL, https://www.wolfram.com/mathematica/.
